# Social epidemiology of early adolescent nutrition

**DOI:** 10.1038/s41390-025-03838-z

**Published:** 2025-01-27

**Authors:** Jason M. Nagata, Christiane K. Helmer, Jennifer Wong, Thang Diep, Sydnie K. Domingue, Richard Do, Ruthie Ervin, Arjun S. Mehta, Abubakr A. A. Al-shoaibi, Holly C. Gooding, Kyle T. Ganson, Alexander Testa, Fiona C. Baker, Andrea K. Garber

**Affiliations:** 1 Department of Pediatrics, University of California, San Francisco, San Francisco, CA USA; 2 Division of General Pediatrics and Adolescent Medicine, Department of Pediatrics, Emory University School of Medicine, Atlanta, GA USA; 3 Factor-Inwentash Faculty of Social Work, University of Toronto, Toronto, ON Canada; 4 Department of Management, Policy and Community Health, University of Texas Health Science Center at Houston, Houston, TX USA; 5 Center for Health Sciences, SRI International, Menlo Park, CA USA; 6 School of Physiology, University of the Witwatersrand, Johannesburg, South Africa

## Abstract

**Background:**

This study aimed to investigate associations between sociodemographic factors and dietary intake among a diverse population of early adolescents ages 10–13 years in the United States.

**Methods:**

We examined data from the Adolescent Brain Cognitive Development (ABCD) Study in Year 2 (2018–2020, ages 10–13 years, *N* = 10,280). Multivariable linear regression models were conducted to estimate the adjusted associations between sociodemographic factors (age, sex, race and ethnicity, household income, parental education) and dietary intake of various food groups, measured by the Block Kids Food Screener.

**Results:**

Older age among early adolescents was associated with slightly less fruit, whole grain, and dairy and more monounsaturated fat consumption. Male sex was associated with a lower intake of fruit, fruit juice, vegetables, whole grains, and fiber and a higher intake of meat/poultry/fish, added sugars, fat, as well as higher glycemic index and glycemic load compared to female sex. Racial and ethnic minority status, lower household income, and lower parental education were generally associated with less fruit and vegetable consumption and more added sugars.

**Conclusion:**

These findings can guide public health interventions to reduce diet quality disparities by targeting key populations and addressing differences according to socioeconomic status, sex, and race.

**Impact:**

- Sociodemographic disparities in diet quality have been studied, but none have explored sociodemographic associations with specific food groups and components (e.g., different types of fat) in early adolescence.
- In this demographically diverse sample of 10–13-year-old early adolescents in the US, we found sociodemographic disparities in dietary intake across various food groups.
- Most notably, male sex, racial and ethnic minority status, lower household income, and lower parental education were associated with less fruit and vegetable consumption and more added sugars.

## Introduction

Adolescence is a critical period of brain and body development during which food preferences emerge and establish the foundation for adulthood.^1^ As such, the development of poor eating habits and behaviors can play a crucial role in shaping risks for obesity and chronic diseases.^2^ The United States Department of Agriculture (USDA) Dietary Guidelines for Americans provide recommendations for a nutritious and balanced diet for children and adolescents, focusing on essential food groups, including vegetables, fruits, whole grains, and proteins like seafood, poultry, and nuts and seeds.^3^ However, most adolescents fail to adhere to these recommendations.^4^ In 2020, the Healthy Eating Index (HEI) of children and adolescents aged 2–18 (HEI = 54) was below the total average American population’s (HEI = 58),^5^ indicating poor diet quality.^6^

Social epidemiology^7^ has linked individual health outcomes to demographic factors (e.g., sex, race and ethnicity, and sexual orientation) and socioeconomic status (e.g., income and education).^8^ In the field of nutrition, Black children have lower diet quality than other racial/ethnic groups,^9,10^ low-income households have poor adherence to the USDA Dietary Guidelines,^11^ and lower parental education level has yielded mixed results for children and adolescents aged 4–18.^4,12^ Early adolescence in itself is a risk factor for unhealthy eating behaviors;^1,13^ however, few studies have investigated the social epidemiology of nutrition in this age group. During this developmental stage, early adolescents experience notable cognitive shifts, such as a growing sense of autonomy in decision-making^13^ and an increased dependence on peer acceptance.^14^ In addition to these developmental changes, an adolescent’s environmental context (e.g., household income and parental education level) can significantly influence their engagement in risky health behaviors, including dietary habits and eating patterns.^15^ Additionally, early adolescents eat a majority of their calories at home,^16^ signaling the importance of family and home environments. To support the nutritional demands required for pubertal growth in early adolescence, it is essential to understand the sociodemographic factors shaping their diets during this developmental period. Previous studies using nationally representative data often combined children and adolescents aged 2–19 together when investigating the association between sociodemographic factors and specific food groups or stratified participants by age groups only when calculating dietary quality scores.^4,9,10,12^ These approaches may complicate efforts by clinicians and public policymakers to evaluate specific food patterns and intervene accordingly. Moreover, existing literature used older samples that are now over a decade old^4,9,10^ and generally examined only a limited number of food groups (e.g., processed meat, sugar-sweetened beverages, and whole grains) or components (e.g., added sugars and saturated fats).^9,17,18^ Advances in nutritional science have brought new insights into the nuanced health effects within these larger categories (e.g., saturated, poly-, and monounsaturated fats)^19^ and nutritional concepts (e.g., glycemic load)^20^ that could further guide targeted interventions that foster healthy diets and eating habits in early adolescence.

To address the gaps in the literature, our study aims to determine the association between sociodemographic factors (age, sex, race and ethnicity, household income, parental education) and dietary intake of various food groups from a large, nationally diverse sample of 10–13-year-old early adolescents in the United States (US).

## Methods

### Study population

We examined data from the Adolescent Brain Cognitive Development (ABCD) study in Year 2 (2018–2020), with participants aged 10–13 years. The ABCD study is an ongoing longitudinal study that tracks brain development, cognitive growth, and adolescent health and recruited 11,875 children from 21 sites across the US in 2016 (baseline). We excluded participants who had missing data for sociodemographic characteristics (*n* = 1365) or non-representative daily energy intakes (<500 kcal/day)^21^ based on the Block Kids Food Screener (BKFS) (*n* = 317), yielding a total of 10,280 participants for our analysis (Appendix A). The study received approval from the Institutional Review Board at the University of California, San Diego, as well as each study site. Caregivers provided informed written consent, and each child gave written assent.

### Sociodemographic factors

Parents/caregivers were asked to provide sociodemographic information about their children, including age, child’s sex assigned at birth (male or female), race and ethnicity (Asian, Black, Latino/Hispanic, Native American, White, or other), annual household income (six categories), and the highest level of parental education (high school diploma or less, or college degree or higher).

### Dietary intake—Block Kids Food Screener

The BKFS is a two-page, 41-item food frequency questionnaire developed by NutritionQuest to evaluate dietary intake of nutrients in food groups in children aged 2–17 years.^22^ BKFS has been validated for estimating food group and added sugar intake but not total energy intake.^22^ The BKFS was validated using three 24-h Nutrition Data System for Research dietary recalls as a reference; the correlations found ranged from 0.5 to 0.9, indicating good relative validity.^22^ Parents/caregivers reported the frequency and quantity of foods and beverages their child consumed over the previous week, which was subsequently reviewed by their child. Our study used parent-reported BKFS data in Year 2.

Summary data from BKFS was provided by NutritionQuest^22^ and included estimated average daily intakes of various food categories: ounce/cup equivalents of fruit/fruit juice, vegetables, potatoes (including french fries), whole grains, saturated fat, meat/poultry/fish, dairy, legumes, and added sugars (tsp). The USDA MyPyramid Equivalents Database (MPED) 2.0 defined the servings for each food group.^3^ The MPED indicates how many MyPyramid equivalents are present in 100 grams (g) of each food consumed by NHANES participants.^3^ For adequate comparison of participant dietary intake, we converted food categories measured in ounces or teaspoons to grams and standardized dietary intake per 1000 kcal (4128 kJ), as done in previous BKFS studies.^23,24^ Specifically, we divided each participant’s dietary intake of food groups and added sugars by their total energy intake and multiplied by 1000 kcal. The standardized intake of food groups and added sugars was referenced against the recommended intakes from the 2020–2025 Dietary Guidelines for Americans for children aged 9–13 years.^3^ All the BKFS food categories were assessed based on the USDA MyPyramid equivalents. We separated the fruit/fruit juice category for a more nuanced assessment by converting the fruit juice variable from gram to cup equivalents and then subtracting this value from the fruit/fruit juice cup equivalents variable.

In addition to BKFS food categories based on the USDA MyPyramid equivalents, summary estimates for glycemic index (glucose scale), glycemic load (glucose scale), total fiber (g), and total energy (kcal) were assessed. In addition to total saturated fat, which was a predetermined BKFS food category, we also examined harmful saturated fat (sum of lauric, myristic, palmitic, stearic acid [g]), monounsaturated fat (sum of palmitoleic and oleic acid [g]), and polyunsaturated fats (PUFAs) ratio (omega-6/omega-3 = [linoleic acid + arachidonic acid]/[alpha-linoleic acid + eicosapentaenoic acid + docosahexaenoic acid]).

Overall, the dependent variables assessed by BKFS were fruit, fruit juice, vegetables, potatoes (including french fries), whole grains, fat (total saturated fat, harmful saturated fat, PUFAs ratio), meat/poultry/fish, dairy, legumes, added sugars, fiber, average daily glycemic index, and average daily glycemic load.

### Statistical analysis

Data analyses for this study were performed in 2024 using Stata 18 (StataCorp, College Station, TX). Descriptive statistics were calculated by using the mean and standard deviation for sociodemographic factors and food groups that were mostly approximately normally distributed, and percentages for sociodemographic factors. Multiple multivariable linear regression models were conducted to estimate the cross-sectional associations between sociodemographic factors (age, sex, race and ethnicity, household income, and parental education) and intake of various food groups. Models were adjusted for sociodemographic factors and study site. Sampling weights based on the American Community Survey were used to represent population estimates.^25^ Statistical significance was based on a two-sided *p* < 0.05.

## Results

Of our adolescent sample (*N* = 10,280) in Year 2, the mean age was 11.6 (±0.7) years, 47.9% of the participants were female, 45.0% were racial and ethnic minorities, and 48.2% came from a household with an annual income of less than $75,000, the approximate median income in the US (Table 1). Table 2 presents a summary of daily dietary intakes in our sample and the recommended intake for children 9–13 years based on USDA Dietary Guidelines for Americans 2020–2025^3^ (Table 2). Table 3 presents the adjusted linear regression models that analyze the association between sociodemographic factors and the intake of various food groups. Effect sizes for associations are based on a change of cups or g per day per 1000 kcal, but we will omit “per 1000 kcal” in our results summary for readability.

**Table 1:** Sociodemographic characteristics of 10,280 Adolescent Brain Cognitive Development (ABCD) Study participants in Year 2 (2018–2020).

Sociodemographic characteristics	Mean (SD)/(%)
Age (years)	11.6 (0.7)
Sex	
Female	47.9%
Male	52.1%
Race and ethnicity	
Asian	5.5%
Black	16.1%
Latino/Hispanic	19.0%
Native American	3.0%
Other	1.4%
White	55.0%
Household income	
$24,999 or less	14.8%
$25,000–$49,999	17.5%
$50,000–$74,999	15.9%
$75,000–$99,999	14.0%
$100,000–$199,999	28.0%
$200,000 or greater	9.8%
Parent’s highest education	
High school education or less	12.6%
College education or more	87.4%

Sampling weights were applied to yield representative estimates based on the American Community Survey from the US Census.

*SD* standard deviation.

**Table 2:** Daily dietary intakes from the Block Kids Last Week Food Screener of 10,280 Adolescent Brain Cognitive Development (ABCD) Study participants in Year 2 (2018–2020).

	Study sample		Recommended^a^
Food group	Mean (SD)	Mean (SD) per 1000 kcals	Per 1000 kcals
Fruit (cups/day)	0.8 (0.7)	0.7 (0.6)	0.94^b^
Fruit juice (cups/day)	0.4 (0.5)	0.3 (0.4)	
Vegetables (cups/day)	0.7 (0.4)	0.6 (0.3)	0.80
Potatoes (cups/day)	0.3 (0.2)	0.2 (0.1)	0.36^c^
Whole grains (g/day)	14.7 (12.3)	12.0 (9.0)	53.16
Fat			
Total saturated fat (g/day)	18.3 (8.2)	14.7 (2.3)	<11.1
Harmful saturated fat (g/day)	16.3 (7.3)	13.0 (2.0)	N/A
Monounsaturated fat (g/day)	17.0 (7.7)	13.6 (2.0)	N/A
Polyunsaturated fats ratio	9.3 (1.6)	–	N/A
Meat, poultry, fish (g/day)	71.9 (43.9)	57.3 (23.0)	78.47
Dairy (cups/day)	1.4 (0.8)	1.2 (0.5)	1.88
Legumes (cups/day)	0.1 (0.1)	0.1 (0.1)	0.09
Added Sugar (g/day)	30.1 (19.7)	24.1 (11.7)	<25.0
Fiber (g/day)	10.7 (4.9)	8.8 (2.8)	13.75 (F), 15.63 (M)
Average daily glycemic index (glucose scale)	50.5 (3.7)	–	N/A
Average daily glycemic load (glucose scale)	68.6 (29.1)	–	N/A
Energy, kcal/day	1241.2 (504.4)	–	1400–2200 (F), 1600–2600 (M)

Sampling weights based on the American Community Survey were used to represent population estimates.

*SD* standard deviation.

^a^Recommended intakes per 1000 kcals are based on USDA Dietary Guidelines for Americans 2020–2025 recommended average daily intake amounts for 1600 kcal level for ages 9–13.

^b^Recommended total fruit and fruit juice intake is based on USDA Dietary Guidelines for Americans 2020–2025 for fruit, including fresh, frozen, canned, and dried fruits and 100% fruit juices.

^c^Recommended potatoes intake is based on USDA Dietary Guidelines for Americans 2020–2025 for starchy vegetables since the vast majority of starchy vegetables consumed by the US population is in the form of white potatoes.

**Table 3:** Sociodemographic associations with daily dietary intakes of 10,280 Adolescent Brain Cognitive Development (ABCD) Study participants in Year 2 (2018–2020).

	Fruit (cups/day) per 1,000 kcals		Fruit juice (cups/day) per 1,000 kcals		Vegetables (cups/day) per 1,000 kcals		Potatoes (cups/day) per 1,000 kcals		Whole Grains (g/day) per 1,000 kcals		Total saturated fat (g/day) per 1,000 kcals		Harmful saturated fat (g/day) per 1,000 kcals		Monounsaturated fat (g/day) per 1,000 kcals	
Sociodemographic characteristics	Adjusted B (95% CI)	p	Adjusted B (95% CI)	p	Adjusted B (95% CI)	p	Adjusted B (95% CI)	p	Adjusted B (95% CI)	p	Adjusted B (95% CI)	p	Adjusted B (95% CI)	p	Adjusted B (95% CI)	p
Age	**-0.03 (-0.05, -0.02)**	**<0.001**	0.01 (0.00, 0.02)	0.250	0.01 (0.00, 0.02)	0.227	**0.01 (0.00, 0.01)**	**0.010**	**-0.39 (-0.67, -0.10)**	**0.008**	0.03 (-0.04, 0.10)	0.454	0.03 (-0.03, 0.10)	0.294	**0.10 (0.04, 0.16)**	**0.002**
Sex																
Female	reference		reference		reference		reference		reference		reference		reference		reference	
Male	**-0.14 (-0.16, -0.12)**	**<0.001**	**-0.03 (-0.04, -0.01)**	**0.001**	**-0.08 (-0.09, -0.06)**	**<0.001**	**-0.01 (-0.02, -0.01)**	**<0.001**	**-0.46 (-0.84, -0.08)**	**0.019**	**0.35 (0.25, 0.44)**	**<0.001**	**0.26 (0.17, 0.34)**	**<0.001**	**0.48 (0.40, 0.57)**	**<0.001**
Race and ethnicity																
Asian	0.07 (-0.01, 0.14)	0.069	-0.01 (-0.04, 0.03)	0.711	**0.05 (0.01, 0.10)**	**0.017**	**0.03 (0.01, 0.05)**	**0.001**	**-3.04 (-3.99, -2.09)**	**<0.001**	-0.08 (-0.35, 0.18)	0.542	0.01 (-0.22, 0.24)	0.941	0.20 (-0.01, 0.41)	0.067
Black	**-0.08 (-0.12, -0.05)**	**<0.001**	**0.20 (0.17, 0.23)**	**<0.001**	**-0.05 (-0.07, -0.03)**	**<0.001**	**0.01 (0.00, 0.02)**	**0.027**	**-0.58 (-1.17, 0.01)**	**0.053**	**-1.00 (-1.15, -0.85)**	**<0.001**	**-0.79 (-0.92, -0.66)**	**<0.001**	0.11 (-0.02, 0.25)	0.104
Latino/Hispanic	-0.01 (-0.05, 0.03)	0.521	**0.11 (0.08, 0.13)**	**<0.001**	**-0.03 (-0.06, -0.01)**	**0.003**	0.00 (-0.01, 0.01)	0.938	**-1.54 (-2.18, -0.90)**	**<0.001**	**-0.61 (-0.78, -0.44)**	**<0.001**	**-0.48 (-0.63, -0.34)**	**<0.001**	**-0.19 (-0.33, -0.04)**	**0.012**
Native American	**-0.07 (-0.13, -0.01)**	**0.024**	**0.05 (0.00, 0.10)**	**0.047**	0.02 (-0.02, 0.06)	0.409	0.02 (0.00, 0.03)	0.056	-0.20 (-1.50, 1.09)	0.760	**-0.41 (-0.72, -0.09)**	**0.011**	-0.28 (-0.56, 0.00)	0.050	0.08 (-0.20, 0.36)	0.559
Other	0.14 (-0.04, 0.32)	0.133	**0.10 (0.01, 0.19)**	**0.030**	0.06 (-0.02, 0.14)	0.114	0.02 (-0.02, 0.06)	0.337	0.69 (-2.80, 4.17)	0.700	**-0.79 (-1.34, -0.23)**	**0.005**	**-0.59 (-1.08, -0.09)**	**0.020**	-0.16 (-0.69, 0.36)	0.545
White	reference		reference		reference		reference		reference		reference		reference		reference	
Household income																
$24,999 or less	**-0.17 (-0.22, -0.12)**	**<0.001**	**0.09 (0.06, 0.12)**	**<0.001**	**-0.05 (-0.08, -0.02)**	**0.002**	**0.03 (0.02, 0.04)**	**<0.001**	**-1.96 (-2.77, -1.14)**	**<0.001**	0.10 (-0.11, 0.31)	0.352	0.16 (-0.02, 0.35)	0.075	**0.42 (0.24, 0.60)**	**<0.001**
$25,000 to $49,999	**-0.20 (-0.25, -0.15)**	**<0.001**	**0.08 (0.05, 0.11)**	**<0.001**	**-0.07 (-0.09, -0.04)**	**<0.001**	**0.03 (0.01, 0.04)**	**<0.001**	**-1.3 (-2.10, -0.50)**	**0.001**	0.19 (-0.02, 0.39)	0.071	**0.23 (0.05, 0.41)**	**0.012**	**0.44 (0.27, 0.61)**	**<0.001**
$50,000 to $74,999	**-0.18 (-0.23, -0.13)**	**<0.001**	**0.04 (0.01, 0.07)**	**0.003**	**-0.06 (-0.09, -0.03)**	**<0.001**	**0.03 (0.02, 0.04)**	**<0.001**	**-1.37 (-2.15, -0.59)**	**0.001**	0.15 (-0.05, 0.34)	0.139	**0.20 (0.03, 0.36)**	**0.022**	**0.53 (0.37, 0.69)**	**<0.001**
$75,000 to $99,999	**-0.13 (-0.18, -0.08)**	**<0.001**	**0.05 (0.02, 0.07)**	**<0.001**	**-0.05 (-0.08, -0.03)**	**<0.001**	**0.02 (0.01, 0.03)**	**<0.001**	-0.52 (-1.27, 0.23)	0.178	0.11 (-0.08, 0.30)	0.275	0.13 (-0.03, 0.29)	0.123	**0.27 (0.11, 0.42)**	**0.001**
$100,000 to $199,999	**-0.10 (-0.14, -0.06)**	**<0.001**	**0.03 (0.01, 0.05)**	**0.004**	**-0.04 (-0.06, -0.01)**	**0.001**	**0.02 (0.01, 0.03)**	**<0.001**	0.01 (-0.63, 0.65)	0.982	0.10 (-0.05, 0.26)	0.195	0.12 (-0.02, 0.25)	0.082	**0.23 (0.11, 0.36)**	**<0.001**
$200,000 or greater	reference		reference		reference		reference		reference		reference		reference		reference	
Parent's highest education																
High school education or less	**-0.04 (-0.08, -0.01)**	**0.019**	0.02 (-0.01, 0.05)	0.145	**-0.06 (-0.08, -0.04)**	**<0.001**	0.00 (-0.01, 0.01)	0.597	-0.47 (-1.13, 0.19)	0.163	**-0.20 (-0.37, -0.03)**	**0.023**	-0.14 (-0.29, 0.01)	0.059	-0.07 (-0.23, 0.09)	0.402
College education or more	reference		reference		reference		reference		reference		reference		reference		reference	

	Polyunsaturated fats ratio		Meat, poultry, fish (cups/day) per 1,000 kcals		Dairy (cups/day) per 1,000 kcals		Legumes (g/day) per 1,000 kcals		Added sugar (g/day) per 1,000 kcals		Glycemic index (glucose scale)		Glycemic load (glucose scale)		Fiber (g/day) per 1,000 kcals	
Sociodemographic characteristics	Adjusted B (95% CI)	p	Adjusted B (95% CI)	p	Adjusted B (95% CI)	p	Adjusted B (95% CI)	p	Adjusted B (95% CI)	p	Adjusted B (95% CI)	p	Adjusted B (95% CI)	p	Adjusted B (95% CI)	p
Age	0.00 (-0.05, 0.05)	0.961	0.68 (-0.06, 1.43)	0.072	**-0.02 (-0.04, -0.01)**	**0.004**	**0.00 (0.00, 0.01)**	**0.007**	0.36 (-0.26, 0.97)	0.254	0.05 (-0.07, 0.17)	0.378	0.08 (-0.82, 0.98)	0.859	-0.07 (-0.16, 0.01)	0.096
Sex																
Female	reference		reference		reference		reference		reference		reference		reference		reference	
Male	**0.14 (0.07, 0.21)**	**<0.001**	**3.07 (2.07, 4.08)**	**<0.001**	0.01 (-0.02, 0.03)	0.668	**0.01 (0.00, 0.01)**	**0.001**	**5.28 (4.45, 6.11)**	**<0.001**	**0.23 (0.07, 0.39)**	**0.006**	**9.40 (8.21, 10.59)**	**<0.001**	**-0.76 (-0.88, -0.65)**	**<0.001**
Race and ethnicity																
Asian	**-0.28 (-0.46, -0.11)**	**0.002**	**5.90 (2.84, 8.96)**	**<0.001**	0.00 (-0.06, 0.07)	0.978	-0.01 (-0.02, 0.00)	0.086	**-5.18 (-6.83, -3.53)**	**<0.001**	-0.41 (-0.87, 0.04)	0.074	**-6.60 (-9.14, -4.06)**	**<0.001**	-0.20 (-0.56, 0.17)	0.287
Black	**0.32 (0.21, 0.42)**	**<0.001**	**2.51 (0.98, 4.04)**	**0.001**	**-0.29 (-0.32, -0.26)**	**<0.001**	**-0.01 (-0.01, -0.01)**	**<0.001**	**4.76 (3.19, 6.34)**	**<0.001**	**0.95 (0.70, 1.20)**	**<0.001**	**12.87 (10.55, 15.18)**	**<0.001**	**-0.50 (-0.67, -0.34)**	**<0.001**
Latino/Hispanic	-0.09 (-0.21, 0.03)	0.134	1.70 (-0.01, 3.41)	0.052	**-0.10 (-0.14, -0.06)**	**<0.001**	**0.04 (0.03, 0.04)**	**<0.001**	**-1.44 (-2.83, -0.06)**	**0.041**	-0.28 (-0.58, 0.01)	0.062	-0.85 (-2.90, 1.19)	0.413	0.16 (-0.04, 0.36)	0.114
Native American	0.03 (-0.18, 0.24)	0.775	2.18 (-0.89, 5.25)	0.164	**-0.10 (-0.17, -0.04)**	**0.002**	0.01 (-0.00, 0.02)	0.211	0.20 (-2.39, 2.79)	0.878	0.02 (-0.49, 0.53)	0.950	3.46 (-0.45, 7.36)	0.083	0.01 (-0.33, 0.36)	0.937
Other	-0.17 (-0.63, 0.28)	0.458	0.61 (-4.54, 5.76)	0.816	**-0.20 (-0.32, -0.08)**	**0.001**	**-0.02 (-0.03, -0.00)**	**0.021**	-4.85 (-9.98, 0.29)	0.064	0.22 (-0.89, 1.33)	0.698	**-6.07 (-11.96, -0.17)**	**0.044**	0.40 (-0.28, 1.08)	0.246
White	reference		reference		reference		reference		reference		reference		reference		reference	
Household income																
$24,999 or less	-0.08 (-0.22, 0.07)	0.318	**2.68 (0.48, 4.88)**	**0.017**	**-0.06 (-0.11, -0.01)**	**0.019**	0.00 (-0.01, 0.01)	0.831	**3.92 (2.08, 5.76)**	**<0.001**	0.22 (-0.09, 0.53)	0.165	**-5.47 (-8.12, -2.82)**	**<0.001**	**-0.76 (-1.02, -0.51)**	**<0.001**
$25,000 to $49,999	0.01 (-0.13, 0.16)	0.847	1.78 (-0.35, 3.90)	0.102	-0.05 (-0.10, 0.00)	0.054	0.00 (-0.01, 0.00)	0.165	**1.62 (0.08, 3.16)**	**0.039**	**0.38 (0.05, 0.71)**	**0.024**	**-5.36 (-8.05, -2.67)**	**<0.001**	**-0.86 (-1.11, -0.61)**	**<0.001**
$50,000 to $74,999	0.06 (-0.07, 0.20)	0.358	**2.66 (0.61, 4.72)**	**0.011**	**-0.08 (-0.13, -0.03)**	**0.001**	**-0.01 (-0.01, 0.00)**	**0.027**	**2.21 (0.75, 3.67)**	**0.003**	0.10 (-0.22, 0.43)	0.533	**-5.46 (-8.10, -2.82)**	**<0.001**	**-0.77 (-1.00, -0.53)**	**<0.001**
$75,000 to $99,999	0.11 (-0.02, 0.25)	0.098	0.15 (-1.84, 2.13)	0.884	-0.05 (-0.10, 0.00)	0.059	0.00 (-0.01, 0.01)	0.721	**1.63 (0.32, 2.95)**	**0.015**	0.23 (-0.05, 0.52)	0.112	**-5.27 (-7.74, -2.80)**	**<0.001**	**-0.41 (-0.65, -0.17)**	**0.001**
$100,000 to $199,999	0.07 (-0.05, 0.18)	0.239	0.89 (-0.78, 2.56)	0.297	**-0.04 (-0.08, -0.00)**	**0.039**	0.00 (-0.01, 0.00)	0.173	**1.16 (0.08, 2.24)**	**0.035**	-0.05 (-0.38, 0.27)	0.746	**-6.24 (-8.87, -3.61)**	**<0.001**	**-0.30 (-0.50, -0.10)**	**0.003**
$200,000 or greater	reference		reference		reference		reference		reference		reference		reference		reference	
Parent's highest education																
High school education or less	0.01 (-0.11, 0.13)	0.856	-1.41 (-3.21, 0.38)	0.123	-0.01 (-0.04, 0.03)	0.786	**0.01 (0.00, 0.01)**	**0.031**	**5.05 (3.27, 6.83)**	**<0.001**	0.14 (-0.16, 0.44)	0.353	**7.72 (5.06, 10.38)**	**<0.001**	**-0.22 (-0.42, -0.02)**	**0.030**
College education or more	reference		reference		reference		reference		reference		reference		reference		reference	

**Bold** indicates p<0.05. B=coefficient from linear regression model. Models represent the abbreviated output from the linear regression models with adjustment for age, sex, race and ethnicity, household income, and parent education.

B coefficient from linear regression model.

### Age

Older age was associated with fewer cups/day of fruit (coefficient [*B*] = −0.03; 95% confidence interval [CI] −0.05, −0.02), fewer g/day of whole grains (*B* = −0.39; 95% CI −0.67, −0.10), more g/day of monounsaturated fat (*B* = 0.10; 95% CI 0.04, 0.16), and less cups/day of dairy (*B* = −0.02, 95% CI −0.04, −0.01) (Table 3).

### Sex

Male sex was associated with fewer cups/day of fruit (*B* = −0.14; 95% CI −0.16, −0.12), cups/day of fruit juice (*B* = −0.03; CI −0.04, −0.01), cups/day of vegetables (*B* = −0.08; 95% CI −0.09, −0.06), and g/day of whole grains (*B* = −0.46; CI −0.84, −0.08) compared to female sex. Male sex was also associated with more cups/day of meat/poultry/fish (*B* = 3.07; 95% CI 2.07, 4.08), g/day of total saturated fat (*B* = 0.35; CI 0.25, 0.44), harmful saturated fat (*B* = 0.26; CI 0.17, 0.34), monounsaturated fat (*B* = 0.48; CI 0.40, 0.57) as well as a higher PUFAs ratio (*B* = 0.14; CI 0.07, 0.21) compared to female sex. Additionally, male sex was associated with more g/day of added sugars (*B* = 5.28; 95% CI 4.45, 6.11) and a higher glycemic index (*B* = 0.23; CI 0.07, 0.39) and glycemic load (*B* = 9.40; CI 8.21, 10.59) compared to female sex. Furthermore, male sex was associated with fewer g/day of fiber (*B* = −0.76; 95% CI −0.88, −0.65) compared to female sex (Table 3).

### Race and ethnicity

Compared to White adolescents, Black and Native American adolescents consumed less fruit, and all racial and ethnic groups except Asian consumed more fruit juice. Black and Latino/Hispanic adolescents consumed less vegetables, and Asian adolescents consumed more vegetables than their White counterparts. Asian and Black adolescents consumed more potatoes (including french fries) compared to their White counterparts. Asian and Latino/Hispanic adolescents had lower intake of whole grains compared to White adolescents.

Compared to White race, all racial and ethnic groups except Asian were associated with less total saturated fat, and Black, Latino/Hispanic, and other race and ethnicity were associated with lower intake of harmful saturated fat. Furthermore, Latino/Hispanic adolescents had lower intake of monounsaturated fat compared to White adolescents. Compared to White adolescents, Asian adolescents had a lower polyunsaturated fatty acids (PUFAs) ratio, while Black adolescents had a higher PUFAs ratio. Asian and Black adolescents had a higher intake of meat/poultry/fish than White adolescents. All racial and ethnic groups except Asian had lower dairy intake compared to White adolescents. Latino/Hispanic adolescents had more grams of legumes than White adolescents, while Black adolescents and those of other race had lower intake of legumes.

Asian and Latino/Hispanic adolescents consumed lower amounts of added sugars compared to White adolescents, while Asian adolescents and those of other races had a lower glycemic load than their White counterparts. Black adolescents had less fiber, more added sugar, higher glycemic index, and a higher glycemic load compared to White adolescents. (Table 3).

### Household income

Lower household income was associated with lower intake of fruit, vegetables, whole grains, dairy, and fiber compared to a household income of $200,000 or greater. Lower household income was associated with a higher intake of fruit juice, potatoes (including french fries), harmful saturated fat, monounsaturated fat, meat/poultry/fish, and added sugars compared to a household income of $200,000 or greater. Additionally, lower household income was associated with a lower glycemic load compared to having a household income of $200,000 or greater (Table 3).

### Parent’s highest education

Adolescents whose parents had an education level of high school or less were associated with fewer cups/day of fruit (*B* = −0.04; 95% CI −0.08, −0.01) and vegetables (*B* = −0.06; 95% CI −0.08, −0.04), fewer g/day of total saturated fat (*B* = −0.20; 95% CI −0.37, −0.03), higher g/day of added sugars (*B* = 5.05; 95% CI 3.27, 6.83), higher glycemic load (*B* = 7.72; 95% CI 5.06, 10.38), and lower g/day of fiber (*B* = −0.22; 95% CI −0.42, −0.02) compared to parents with a college education or more (Table 3).

## Discussion

### Summary of findings

In our demographically diverse sample of 10–13-year-old early adolescents in the US, we found that the sociodemographic factors that were generally associated with less healthy nutrition patterns were older age, male sex, racial and ethnic minority status, lower household income, and lower parental education. Particularly, older age was associated with less fruit, whole grain, and dairy consumption and more monounsaturated fat consumption. Male sex was associated with a higher intake of meat/poultry/fish, added sugars, and fats and lower intake of fiber-rich foods compared to female sex. Racial and ethnic minority status, lower household income, and lower parental education were associated with more added sugars and less fruit and vegetable consumption.

### Age

Older age among early adolescents was associated with slightly less fruit, whole grain, and dairy consumption and more monounsaturated fat consumption. While our findings were of small effect size, they align with current literature showing diet quality tends to decrease as a child gets older.^9,12^ Potential explanations for this inverse relationship include increased marketing to this age group and more autonomy and opportunities to select processed and less nutritious food.^9,17^

### Sex

Our study found that male early adolescents had a lower-quality diet with less fiber, fruit, fruit juice, vegetables, whole grains, and higher glycemic index and load than their female counterparts. This dietary pattern is consistent with prior research in adults showing that women have better-quality diets than men.^26–29^ Gender norms and roles may play a role in shaping these dietary choices by encouraging health consciousness and body image obsession among women.^30,31^ Research on body weight and dieting also shows that women across all age groups reported more worries about weight and took more action to control weight,^32,33^ which may involve low-calorie diets consisting of more fruits and vegetables.

Male early adolescents also consumed more meat, poultry, and/or fish compared to female early adolescents. Since participants in our study are aged 10–13, a period when male puberty typically begins, our findings may reflect differences in the nutritional demands of male adolescents during this developmental stage.^34^ Consumption of food with higher protein content like meat, poultry, and/or fish may also be explained by the societal pressures for men to pursue a muscular physique.^35^ The desire for muscularity may involve dietary modifications aimed at enhancing muscle mass such as overregulation of protein consumption and elimination of non-protein-based foods.^36^

Our study also showed that male early adolescents consumed more fat, including total saturated fat, harmful saturated fat, and healthier monounsaturated fat. Earlier research on fat intake among children and adolescents aged 2–19 found minimal variation in the percentage of energy from fat and saturated fatty acids by sex, with overall averages of 33.5% of energy from fat and 12.2% from saturated fatty acids.^37–39^ However, this data is over two decades old and may not reflect current adolescent diets.^37^ In our study, male adolescents had a higher PUFAs ratio, or omega-6 to omega-3 fatty acid ratio, compared to female adolescents. One study of adolescents aged 12–17 years in Brazil did not observe any sex differences in fatty acid intakes.^40^ With omega-6 fatty acids implicated in various inflammatory responses,^41^ future research should further investigate the relationship between fat consumption and sex differences in early adolescents. Our study adds to the current research by distinguishing various subtypes of fat and highlighting the differences in intake based on sex, with a focus on early adolescents.

Moreover, our study revealed male early adolescents consumed more added sugars and food with a higher glycemic load and glycemic index. This is consistent with prior research showing that boys aged 2–19 years had a higher mean of added sugar intake than girls,^42^ with sugar-sweetened beverages being the leading source of added sugars among high consumers.^43^ Awareness of health risks may explain the sex differences in added sugar intake observed in our study. A separate study on adolescents reported increased odds of drinking sugar-sweetened beverages among those unaware of health risks, with boys being less likely to recognize the risks of cavities and heart disease.^44^

### Race and ethnicity

Consumption of fruit and vegetables was lower among Black and Latino/Hispanic early adolescents in our study compared to their White counterparts. Black adolescents also had fewer g/day of fiber. This aligns with previous research indicating that Black youth tend to have the lowest dietary quality when compared to other racial/ethnic groups.^9,10^ Fruit juice consumption in our study was higher among Black, Latino/Hispanic, Native American, and other racial/ethnic groups compared to White individuals. Since healthy food often comes at a higher cost^45^ and affordability heavily influences food choices,^46^ individuals may opt for more cost-effective options (e.g., apple and citrus juice for fruit, and potatoes for vegetables).^47,48^

In our study, protein intake was higher among Asian and Black adolescents (for meat, poultry, and fish) and Latino/Hispanic adolescents (for legumes) compared to their White counterparts. A similar cross-sectional study analyzing protein intake trends in the US also reported greater protein consumption among adult Asian and Latino/Hispanic populations.^49^ However, unlike our findings, there were no significant race or ethnicity differences in protein intake in participants aged 2–18 years.^49^ Notably, our results indicated that Asian adolescents had the highest meat, poultry, and fish intake among all racial and ethnic groups in our study, which is consistent with previous research suggesting that Asian adolescents generally consume more protein than other races and ethnicities.^50^ Furthermore, data from NHANES describes how Asian adults obtain approximately 17% of their energy intake from consuming protein.^50^ The observed racial and ethnic differences in protein intake may be explained by cultural dietary habits and consumption patterns. For instance, a study involving 12 to 19-year-olds found that Latino/Hispanic males and females displayed dietary patterns characterized by greater consumption of seafood, and plant proteins, such as legumes.^10^ In contrast, Black participants and those of other race were less likely to consume legumes, while Latino/Hispanic participants were more likely to have higher levels of legume consumption, as beans often serve as a cultural staple of the home environment and diet.^51^ Racial and ethnic differences in diet quality and nutrient intake, influenced by cultural dietary norms and eating patterns, may be linked to varying risks for chronic diseases among different racial and ethnic populations.^52^ Thus, future research should consider examining how acculturation may affect dietary intake among minority groups.

In line with prior research, Black adolescents in our study consumed 4.76 more g/day per 1000 kcals of added sugars and had a higher glycemic load compared to White adolescents. Meanwhile, Asian adolescents consumed less added sugars compared to White adolescents. Studies using nationally representative data showed that the per capita consumption of sugar-sweetened beverages was highest among Black, Mexican American, and non-Mexican Hispanic adolescents aged 12–19 years,^53^ and Black high school students had greater odds of high sugar-sweetened beverages intake (≥3 times/day).^54^ Differences in added sugar intake in our study may reflect unhealthy food advertisements targeted to Black and Hispanic youth,^55^ which has been linked to poor diet quality and metabolic diseases like obesity in children.^56,57^ One study also showed that Black youth expressed less distrust of these advertisements and perceived sugary drinks as less harmful compared to White youth.^58^ However, unlike prior studies, we found that Latino/Hispanic adolescents consumed less added sugars than White adolescents. A recent study using 2015–2018 NHANES data did note that the prevalence of adolescents with high added sugar consumption was significantly greater among both non-Hispanic Black and non-Hispanic White youth compared to Hispanic youth.^43^

Harmful saturated fat intake was lower among Black, Latino/Hispanic, and other race and ethnicity adolescents than White adolescents. Another study found similar results, indicating that Black and Hispanic adolescents aged 12–19 years had higher unsaturated fatty acid to saturated fatty acid ratios.^10^ However, the finding was unexpected given that the food industry disproportionately advertises unhealthy food high in saturated fat to youth from racial and ethnic minority backgrounds.^59^ Research can further explore the underlying factors shaping the consumption of different subtypes of fat in early adolescence. Additionally, our study shows that, compared to White adolescents, Asian adolescents had a lower PUFAs ratio while Black adolescents had a higher ratio. Research using NHANES data from 2011–2014 on adult fatty acid intake found similar patterns, with non-Hispanic Black adults reporting the highest total PUFAs intake and non-Hispanic Asian adults reporting the lowest.^60^ Given that adolescents’ dietary habits for fat intake may be influenced by those of their parents,^61^ the racial differences in fat consumption may be explained by cultural differences in the diets that early adolescents are exposed to at home. Additionally, based on anthropological and epidemiological studies, Western diets have evolved to include high PUFAs ratios that contribute to the pathogenesis of different cardiovascular, inflammatory, and autoimmune diseases.^62^

### Household income

Our study found that adolescents who had parents with lower annual incomes were more likely to have lower-quality diets that included less fruit, vegetables, whole grains, dairy, and fiber but more added sugars and fruit juice. Prior research suggested that there is an association between lower income and lower diet quality, which may be attributed to accessibility and food insecurity.^63^ Food-insecure households may experience lower dietary quality due to inconsistent dietary patterns and failure to meet nutrition guidelines.^63^ Because adolescence is a critical developmental time period where nutrition is an essential component for growth as well as mental and physiological health, low diet quality may predispose adolescents to adverse health outcomes in adulthood.

Lower-income adolescents in our study consumed less fruit, vegetables, and fiber in their diets. Disparities in accessibility may explain the lack of fiber, as income acts as a barrier to consuming fruits and vegetables. Prior research explained that adolescents who met the fiber recommendation guidelines often consumed increased amounts of fruits and vegetables, in comparison to adolescents who failed to meet the recommended amount.^64^ A separate study on nutrient intake in US infants and toddlers found that children with the lowest energy-adjusted fiber intake consumed the highest amounts of food and beverages from sweets and the lowest quantity of fruits.^65^ Because fruit is one of the main sources of fiber intake among US adolescents, low-income households are less likely to consume fruit and, consequently, less fiber compared to higher-income households.

Compared to higher-income adolescents in our study, lower-income adolescents consumed more added sugars. Similarly, a study using NHANES revealed that adolescents from lower-income households consumed increased amounts of added sugars from snacks and beverages in comparison to those from higher-income households.^66^ Prior research has also explained that sugar-sweetened beverages are a major source of added sugars in the diets of adolescents and are linked to adverse health outcomes, such as diabetes, cardiovascular disease risk, and dyslipidemia.^43^ Lower-income households are more likely to consume cheaper, energy-dense foods, such as starches, vegetable fats, and added sugars, contributing to low-cost, low-quality diets.^67^

### Parent’s highest education

Our study found that adolescents whose parents had a high school equivalent education or less were associated with less fruit, vegetables, and fiber, more added sugars, and higher glycemic load. Prior research has found that adolescents with lower socioeconomic status (SES) tend to consume more low-quality foods due to unequal access to a nutrient-dense diet, with parental education being a key factor influencing this trend.^68^ Similar to our findings, one cross-sectional study examining patterns of diet revealed that 10–12-year-old children whose mothers had the lowest levels of education were more likely to consume energy-dense foods and drinks with increased vegetable fats and added sugars.^69^ This may be explained by the relationship between low parental education and low SES, where families of low SES have fewer financial resources to afford healthy, nutrient-rich foods. A study of adolescents from two Norwegian counties found that adolescents from lower SES were associated with lower consumption of fruits and vegetables.^70^ In contrast, adolescents of parents with higher education tend to prefer fruits and vegetables, which may be attributed to their greater knowledge of dietary guidelines and the guidance of role models who encourage nutrient-rich foods and healthy eating habits.^70^

Consistent with our findings on income, adolescents with parents who have lower levels of parental education were associated with higher added sugar intake. A prior study using the NHANES revealed that adolescents with parents who have lower levels of parental education were more likely to consume increased amounts of sugar-sweetened beverages, in comparison to adolescents whose parents had, at least, a college degree.^71^ In fact, it has been suggested that as education level increases, parents have more knowledge of sugar-free awareness.^72^ Parent’s educational level was negatively associated with adolescents’ intake of sweets, sugar-sweetened beverages, and added sugar intake.^73^ This association may be explained by parents from higher educational backgrounds being more likely to use restrictions with unhealthy foods, as well as disparities in accessing nutrient-rich foods in low-income households. Our study contributes to the current literature, supporting that lower parental education worsens diet quality and prevents adequate consumption of nutrient-rich foods due to less access.

### Strengths and limitations

This study has several limitations. This study relied on data reported by parents, which could introduce social desirability bias as they may have under/over-reported dietary intake of some food groups for their child to be perceived more positively.^74^ Furthermore, previous studies comparing the reporting accuracy between parents and children have found that children report food intakes that are closer to their “true” food intake levels.^75,76^ However, in our study, children reviewed their parents’ dietary reports, which helped mitigate this bias and the disconcordance between child and parent questionnaire responses. Additionally, given that the BKFS was primarily designed to assess nutrients and food groups, it has not been validated for measuring total energy intake, leading to underestimates for this measure.^22^ We standardized dietary intake by using the total energy intake for adequate comparison of participant dietary intake, but these standardized estimates should be interpreted with caution when presented on their own. Lastly, our fruit juice calculations have slightly underestimated fruit juice intake and overestimated whole fruit intake because some whole fruit items, such as applesauce/fruit cocktails, have unknown amounts of fruit juice, which was counted toward the whole fruit total rather than the fruit juice total. A notable limitation is a lack of psychosocial influences on eating behaviors (e.g., diet behavior, in-person and online social influences) in our study. A ripe area for future research includes analyzing these psychosocial influences and assessing eating disorders and mood disorders to examine potential influences on eating behaviors. The strengths of this study include the large, demographically diverse sample of 10,280 early adolescents. Additionally, the BKFS is a simple, inexpensive method of assessing dietary intake and has been validated against three 24-h dietary recalls in children aged 10–17 for all food groups and added sugars intake.^22^ Compared to other dietary intake systems, such as the Nutrition Data System for Research (NDSR), which consists of three 24-h days of recalls, the BKFS has reported significantly fewer food group estimates as zero because of the longer reference period of seven days.^22^ Finally, the BKFS reports more ingredients in mixed dishes for some food groups than NDSR, which may allow for more nuanced analyses.^22^

## Conclusion

This study investigated the sociodemographic differences in dietary and nutritional intake behaviors among a diverse population of early adolescents ages 10–13 years in the US. We identified significant disparities in diet quality, with older adolescents, males, racial and ethnic minorities, those from low-income households, and those with lower parental education reporting poorer diet quality. These findings can inform effective public health interventions designed to reduce disparities in diet quality by targeting key populations and considering differences in socioeconomic status, age, sex, and race/ethnicity.^77^ Since early adolescents still consume most of their calories at home, interventions focusing on the family setting—such as providing food education for parents—can improve children’s food literacy, encourage healthy choices, and support good diet quality.^78^ Food marketing and advertising also contribute to poor diet quality among adolescents and teenagers in the US, particularly targeting Black and Latino/Hispanic adolescents with cheaper, energy-dense foods.^79,80^ Clinicians and public health professionals should focus on promoting educational intervention programs that encourage healthy dietary behaviors and protect adolescents from unhealthy food advertising. Future studies should aim to investigate the mechanisms underlying food habits and dietary intake among early adolescents such as screen time and media use.^81^

## Supplementary information

Supplementary information

## Data Availability

Data used in the preparation of this article were obtained from the ABCD Study (https://abcdstudy.org), held in the NIMH Data Archive (NDA).
