# Supplementary material for: Social epidemiology of early adolescent nutrition
Source: Pediatr Res. 2025 Jan 27;98(3):885–94. doi: 10.1038/s41390-025-03838-z (PMC12301997; doi:10.1038/s41390-025-03838-z)
Supplement: Supplementary file 1 — Supplementary information [file 41390_2025_3838_MOESM1_ESM.pdf]

Appendix A. Comparison of the sociodemographic characteristics of the Adolescent Brain Cognitive Development (ABCD) study participants included vs. excluded in the analysis

| Sociodemographic characteristics | Included (n=10,280) | Excluded (n=1,682) | p |
|---|---|---|---|
| Age (years) | 11.6 (0.7) | 11.6 (0.7) | 0.501 |
| Sex | | | <0.001 |
| Female | 47.9% | 54.3% | |
| Male | 52.1% | 45.7% | |
| Race and ethnicity | | | <0.001 |
| Asian | 5.5% | 5.7% | |
| Black | 16.1% | 25.0% | |
| Latino/Hispanic | 19.0% | 26.4% | |
| Native American | 3.0% | 4.1% | |
| Other | 1.4% | 2.0% | |
| White | 55.0% | 36.8% | |
| Household income | | | <0.001 |
| $24,999 or less | 14.8% | 17.9% | |
| $25,000 to $49,999 | 17.5% | 22.0% | |
| $50,000 to $74,999 | 15.9% | 16.0% | |
| $75,000 to $99,999 | 14.0% | 9.5% | |
| $100,000 to $199,999 | 28.0% | 29.2% | |
| $200,000 or greater | 9.8% | 5.4% | |
| Parent's highest education | | | <0.001 |
| High school education or less | 12.6% | 30.0% | |
| College education or more | 87.4% | 70.0% | |
